# Intravenous *γ* Globulin for Intractable Abdominal Pain due to IgA Vasculitis

**DOI:** 10.1155/2020/8867621

**Published:** 2020-10-17

**Authors:** George Naifa, George Totikidis, Sonia Alexiadou, Christina Kolona, Elpis Mantadakis

**Affiliations:** ^1^Resident in Pediatrics, Department of Pediatrics, University General Hospital of Alexandroupolis, Alexandroupoli, Greece; ^2^NHS Registra in Pediatrics, Department of Pediatrics, University General Hospital of Alexandroupolis, Alexandroupoli, Greece; ^3^Pediatrics-Pediatric Hematology/Oncology, Democritus University of Thrace Faculty of Medicine, Alexandroupoli, Thrace, Greece

## Abstract

IgA vasculitis (formerly known as Henoch–Schönlein purpura or anaphylactoid purpura) is a usually benign vasculitis that affects children of school age. The disease is characterized by the tetrad of palpable purpura, arthralgia/arthritis, abdominal pain, and hematuria. Treatment of IgA vasculitis is mainly supportive, with administration of simple analgesics. Corticosteroids have been shown to reduce and/or ameliorate the occurrence of abdominal pain which may be severe. We present two children with IgA vasculitis and severe abdominal pain despite corticosteroid administration, who responded promptly to intravenous *γ* globulin (IVIg) with complete resolution of their symptoms and review of the relevant medical literature. Given the toxicity and/or need for long-term administration of other second-line immunosuppressive therapies in corticosteroid-resistant IgA vasculitis, such as rituximab, cyclosporine, cyclophosphamide, azathioprine, or colchicine, we propose that IVIg may be a useful and safe treatment option, although randomized controlled clinical trials are needed in order to clarify its role in the treatment of abdominal pain in IgA vasculitis.

## 1. Introduction

IgA vasculitis is a systemic vasculitis of small blood vessels [1]. It was formerly known as Henoch–Schönlein purpura, but since 2011, it has been renamed to IgA vasculitis by consensus definition because deposition of IgA in the vessel walls is the prominent histopathological feature of this condition [2]. Despite that, in everyday clinical practice, the term HSP is still commonly used. It is the most common childhood vasculitis with an incidence of 10–20 cases/100,000 children per year [3]. It mainly affects children <10 years of age, with the mean age at presentation being between 4 and 6 years [2]. In IgA vasculitis, immunoglobulin A (IgA) deposition and complement component 3 (C3) activation are observed [4]. IgA vasculitis was initially named after two German physicians, Johann Schönlein and his student Eduard Henoch. Schönlein identified the association of joint pain and purpura, while Henoch identified the gastrointestinal and renal involvement. Although IgA vasculitis was initially named after Henoch and Schönlein, the English physician William Heberden was the first to describe the disorder in the early 1800s [5].

IgA vasculitis is a leukocytoclastic vasculitis that affects small blood vessels and is characterized by the presence of inflammation in the vascular walls resulting in tissue ischemia and necrosis [1]. The disease is also known as anaphylactoid purpura. Ethnicity, gender, and environmental factors are responsible for the different incidence rates of IgA vasculitis in various studies. Caucasians have the highest, and African Americans have the lowest incidence of IgA vasculitis, while men are more frequently affected than women at a ratio of about 2 : 1 [6]. A preceding infection with group A streptococcus, *Staphylococcus aureus*, influenza viruses, Epstein–Barr virus, adenovirus, parvovirus, and *Mycoplasma pneumoniae* have been reported as triggering factors [6].

The main clinical signs and symptoms of IgA vasculitis include purpura, arthralgia, abdominal pain, and hematuria that may or may not occur simultaneously [7, 8]. Criteria for diagnosis of IgA vasculitis include palpable purpuric rash and at least one of the following four: (1) arthritis or arthralgia (82% of the patients), (2) abdominal pain (63%), (3) renal involvement manifested as hematuria and/or proteinuria (up to 40%), and (4) histopathological documentation of IgA deposition [1, 6].

Other clinical features of IgA vasculitis include orchitis, and intussusception. Cardiac, pulmonary, pancreatic, and cerebral involvement is rare. In most cases, the disease lasts for 4 weeks [9] and can occur in an atypical form in which the gastrointestinal signs and symptoms precede the appearance of the skin rash.

We present two children with IgA vasculitis and severe abdominal pain despite corticosteroid administration, who responded promptly to intravenous *γ* globulin (IVIg) administration with complete resolution of their symptoms a few hours after treatment and review of the relevant medical literature.

## 2. Case Presentation

### 2.1. Case 1

A 6-year-old boy, fully immunized for age, was referred to us from a general hospital due to a palpable purpuric skin rash with trouser-like distribution, arthralgias, abdominal pain, and bloody stools. He was afebrile and with the exception of moderate leukocytosis (leukocytes 23,610 *μ*L), mild thrombocytosis (platelets 414,000 *μ*L), and slightly elevated CRP (1.32 mg/dL, normal <0.5), he had normal hemoglobin, 13.7 g/dL; hematocrit, 38.5%; urea, 11 mg/dL; creatinine, 0.4 mg/dL; sodium, 136 mmol/L; potassium, 3.5 mmol/L; AST, 38 U/L; ALT, 31 U/L; prothrombin time (PT), 12.9 sec; activated partial thromboplastin time (aPTT), 24.4 sec; fibrinogen, 312 mg/dL; IgG, 924 mg/dL; IgA, 190 mg/dL; IgM, 102 mg/dL; C3, 138 mg/dL; C4, 29.1 mg/dL; and negative antinuclear antibodies (ANA). Microscopic stool examination demonstrated 50–70 erythrocytes per HPF.

### 2.2. Case 2

An afebrile 3-year-old girl, fully immunized for age, was admitted because of typical IgA vasculitis with purpuric skin rash over the lower extremities. Fewer purpuric lesions were also found on both forearms. In addition, her ankles and elbows were edematous and slightly erythematous, while she had intense abdominal pain and bloody stools. Baseline laboratory studies showed leukocytes, 18,110 *μ*L; hemoglobin, 13.4 g/dL; hematocrit, 39.4%; platelets, 451,000 *μ*L; CRP, 4.7 mg/dL; urea, 24 mg/dL; creatinine, 0.4 mg/dL; sodium, 133 mmoL/L; potassium, 4.5 mmol/L; AST, 26 U/L; ALT, 11 U/L; PT, 14.4 sec; aPTT, 26.9 sec; fibrinogen, 381 mg/dL; IgG, 891 mg/dL; IgA, 210 mg/dL; IgM, 71.4 mg/dL; C3, 125 mg/dL; C4, 33.7 mg/dL; and negative ANA.

In both children, meticulous and repeated physical examinations and abdominal ultrasounds ruled out intussusception, while repeated urinalyses were negative for hematuria and substantial proteinuria. No child was subjected to skin biopsy for immunofluorescence in order to demonstrate dermal IgA staining because their skin lesions were very typical of IgA vasculitis, i.e., both had palpable purpuric rash with typical trouser-like distribution.

In the first child, methylprednisolone was started intravenously at 0.5 mg/kg twice daily. After a period of two days with no symptoms, recurrent and severe abdominal pain occurred to the point that the child was unable to sleep at nighttime without waking up with stabbing periumbilical pain. On the 8^th^ day of hospitalization, he received a single infusion of IVIg at 1 g/kg over 8 hours. He tolerated treatment well. Within a few hours after the administration of IVIg, he had no further recurrences of abdominal pain, which allowed us to gradually taper and finally discontinue the corticosteroids on the 14^th^ day of hospitalization.

The second child initially received intravenous methylprednisolone at 0.5 mg/kg twice daily, while she continued to develop new skin lesions on both shins. Despite the normalization of her bowel movements, on the 9^th^ day of hospitalization due to intractable abdominal pain, she received a single infusion of IVIg at 1 g/kg over 8 hours. After the administration of IVIg, the abdominal pain was relieved within less than a day, and methylprednisolone was gradually tapered and finally discontinued on the 14^th^ day of hospitalization.

Both children were discharged home one day after the discontinuation of corticosteroids, with resolving skin rash and instructions for repeated urinalyses at regular intervals to check for hematuria. They remain asymptomatic, six and four months, respectively, after the described events.

## 3. Discussion

IgA vasculitis, formerly known as anaphylactoid purpura or HSP, is mainly a benign vasculitis that predominantly affects children of school age. The disease is characterized by the tetrad of palpable purpura, arthralgia/arthritis, abdominal pain, and renal involvement. Timing of symptoms may be acute within a day or two or insidious over a period of several weeks. Although it is usually a benign and self-limiting illness, relapses can occur, but the long-term prognosis depends almost exclusively on the severity of renal involvement [4].

Treatment of mild IgA vasculitis is primarily supportive and empirical, with administration of simple analgesics such as paracetamol and nonsteroidal anti-inflammatory drugs [1]. A meta-analysis based on a comprehensive review of the literature (1956 to January 2007) showed that corticosteroids in IgA vasculitis significantly reduce the mean resolution time of abdominal pain, increase the odds of resolution within 24 hours, and significantly reduce the odds of developing persistent renal disease [10]. IgA vasculitis is resistant to corticosteroids, when symptoms persist despite corticosteroid administration for at least 7 days or if recurrence of signs and symptoms occurs after a period of remission despite their continuous administration [4].

For the treatment of severely symptomatic IgA vasculitis, IVIg has been used in the past, albeit rarely [4, 11, 12]. Cherqaoui et al. reported a retrospective case series conducted at 29 French academic pediatric centers of eight children with IgA vasculitis, i.e., five boys and three girls aged 3–15 years (median age 5.5 years) that received IVIg for intense abdominal pain, gastrointestinal bleeding, or protein-losing enteropathy. All children had been treated with corticosteroids as first-line therapy. Six out of eight patients responded completely to IVIg within 7 days, while the remaining two had a partial response. It is of note that two of the eight children relapsed with milder gastrointestinal symptoms and required a second dose of IVIg, with no further recurrences, even though two patients developed high proteinuria on the day following IVIg infusion [4]. In addition, Mauro et al. described an 11-year-old child with IgA vasculitis who presented with severe skin lesions that did not respond to standard therapy with corticosteroids and who was successfully treated with intravenous immunoglobulins [13].

Both of our patients had intense, recurrent, and torturous abdominal pain that was not relieved with pharmacological doses of corticosteroids, and both responded promptly to IVIg, i.e., within a day with complete symptom resolution. Although the dose of IVIg that has been previously used in IgA vasculitis was 2 g/kg of body weight, we used a single infusion of 1 g/kg that is substantially cheaper and likely better tolerated, i.e., associated with a reduced chance of headache and aseptic meningitis [14, 15]. Even though the mechanism of action of IVIg in IgA vasculitis is unknown, immunoglobulin G has anti-inflammatory properties, and in addition, it suppresses the endogenous production of autoantibodies.

We decided to administer IVIg as a single infusion because it has fewer side effects compared to other immunosuppressive therapies that have been used in IgA vasculitis, like rituximab. This humanized monoclonal antibody has been tried in autoantibody-associated vasculitides and in the treatment of chronic steroid-dependent IgA vasculitis and is effective in inducing and maintaining remission in patients with disease resistance to first-line treatment [16].

Our patients did not have renal involvement. It is of note that a systematic review by Zaffanello et al. on the adjuvant treatments of IgA vasculitis with renal involvement did not find any convincing evidence for the efficacy of IVIg in nephritis [17]. Moreover, the use of IVIg has rarely been associated with acute kidney injury in the past [18, 19].

Colchicine is an additional treatment option for IgA vasculitis [20, 21]. A report described two children with prolonged IgA vasculitis including persistent purpuric rash and hematuria that had prompt resolution of their symptoms after colchicine therapy [20]. Allali et al. also reported a single case of pediatric IgA vasculitis with severe cutaneous manifestations that responded well to colchicine [21].

Fotis et al. successfully used azathioprine in six patients with steroid-resistant IgA vasculitis including patients with persistent gastrointestinal symptoms [22]. Cyclosporine has also tried for the treatment of resistant nephritis [23], but its use is associated with several well-known side effects such as hirsutism and profound immunosuppression. Recently, mycophenolate mofetil has gained popularity in the treatment of autoimmune disorders that do not respond to corticosteroids including IgA vasculitis with gastrointestinal symptoms, but no prospective studies exist for this treatment modality in IgA vasculitis [24].

Research suggests that severe gastrointestinal complications in IgA vasculitis are related to the lack of factor XIII, and its administration may be therapeutically helpful [25, 26]. Factor XIII is a transglutaminase that catalyzes the cross-linking of fibrin that plays an important role in hemostasis and wound healing. Several studies have confirmed low plasma factor XIII levels in IgA vasculitis patients with severe abdominal symptoms, but the underlying mechanisms for the decreased factor XIII activity are unknown. Finally, in cases of IgA vasculitis resistant to corticosteroids and IVIg, plasma exchange and leukocytapheresis have been used [27, 28].

## 4. Conclusion

Our report describes the remission of severe abdominal pain in corticosteroid-resistant IgA vasculitis after administration of IVIg. Hence, in IgA vasculitis with severe abdominal pain unresponsive to pharmacological doses of corticosteroids, IVIg should be considered, especially if there is no renal involvement since IVIg is ineffective for IgA vasculitis with renal involvement, i.e., nephritis. Compared to other alternative immunosuppressive therapies, IVIg is likely safer and more convenient, and although costly, it may be cost-effective. Although we used IVIg at 1 g/kg as a single infusion over 8 hours, the proper dose and schedule of IVIg for IgA vasculitis with severe abdominal pain despite administration of corticosteroids remains unknown. Randomized controlled clinical trials comparing different therapeutic options in IgA vasculitis are certainly needed in order to clarify the role of IVIg in the treatment of severe abdominal pain and/or other manifestations of this disease.

## Data Availability

The clinical and laboratory data used to support the findings of this manuscript are included within the article.
